# A Risk Prediction Model by LASSO for Radiation-Induced Xerostomia in Patients With Nasopharyngeal Carcinoma Treated With Comprehensive Salivary Gland–Sparing Helical Tomotherapy Technique

**DOI:** 10.3389/fonc.2021.633556

**Published:** 2021-02-26

**Authors:** Feng Teng, Wenjun Fan, Yanrong Luo, Shouping Xu, Hanshun Gong, Ruigang Ge, Xinxin Zhang, Xiaoning Wang, Lin Ma

**Affiliations:** ^1^ Department of Radiation Oncology, China-Japan Friendship Hospital, Beijing, China; ^2^ Department of Radiation Oncology, Medical School of the Chinese People’s Liberation Army (PLA), Beijing, China; ^3^ Affiliated Foshan Maternity and Child Healthcare Hospital, Southern Medical University, Foshan, China; ^4^ Department of Radiation Oncology, Nanfang Hospital, Southern Medical University, Guangzhou, China; ^5^ Department of Radiation Oncology, Armed Police Corps Hospital of Henan Province, Zhengzhou, China; ^6^ Department of Radiation Oncology, First Medical Center of Chinese PLA General Hospital, Beijing, China; ^7^ Departmant of Otorhinolaryngology, First Medical Center of Chinese PLA General Hospital, Beijing, China; ^8^ School of Data Science and Media Intelligence, Communication University of China, Beijing, China; ^9^ State Key Laboratory of Media Convergence and Communication, Communication University of China, Beijing, China

**Keywords:** xerostomia, nasopharyngeal carcinoma, prediction model, LASSO, helical tomotherapy technique

## Abstract

**Objective:**

This study aimed to develop a least absolute shrinkage and selection operator (LASSO)-based multivariable normal tissue complication probability (NTCP) model to predict radiation-induced xerostomia in patients with nasopharyngeal carcinoma (NPC) treated with comprehensive salivary gland–sparing helical tomotherapy technique.

**Methods and Materials:**

LASSO with the extended bootstrapping technique was used to build multivariable NTCP models to predict factors of patient-reported xerostomia relieved by 50% and 80% compared with the level at the end of radiation therapy within 1 year and 2 years, R50-1year and R80-2years, in 203 patients with NPC. The model assessment was based on 10-fold cross-validation and the area under the receiver operating characteristic curve (AUC).

**Results:**

The prediction model by LASSO with 10-fold cross-validation showed that radiation-induced xerostomia recovery could be predicted by prognostic factors of R50-1year (age, gender, T stage, UICC/AJCC stage, parotid Dmean, oral cavity Dmean, and treatment options) and R80-2years (age, gender, T stage, UICC/AJCC stage, oral cavity Dmean, N stage, and treatment options). These prediction models also demonstrated a good performance by the AUC.

**Conclusion:**

The prediction models of R50-1year and R80-2years by LASSO with 10-fold cross-validation were recommended to validate the NTCP model before comprehensive salivary gland–sparing radiation therapy in patients with NPC.

## Introduction

At present, intensity-modulated radiation therapy (IMRT) combined with chemotherapy is the main treatment model in patients with nasopharyngeal carcinoma (NPC) (1). Radiation-induced xerostomia, as a common and serious adverse effect of radiation therapy (RT), significantly reduces patients’ quality of life, causing difficulties in chewing, swallowing, speaking, and even sleeping patterns (2–4). In recent decades, multiple studies have shown that IMRT could decrease radiation-related xerostomia by sparing parotid glands or submandibular glands (5–7). Nowadays, IMRT technique, especially helical tomotherapy (HT), provides homogeneous dose distribution in target volumes with a low dose to salivary glands. A previous study reported that comprehensive protection of salivary glands, including parotid glands (PGs), submandibular glands (SMGs), and accessory salivary glands in the oral cavity (OC), minimized xerostomia in patients with head and neck cancer (HNC) treated with HT technique, without increasing early locoregional recurrence risk (8).

Xerostomia prediction could assist clinicians to prejudge the probability and severity of this side effect and to design a more suitable treatment plan, if possible, in advance. In recent years, correlations between the probability and severity of xerostomia with irradiation volume and dose to salivary glands were established (9–11). The Quantitative Analyses of Normal Tissue Effects in the Clinic (QUANTEC) guidelines recommended a mean dose (Dmean) below 20 or 25 Gy to one or two PGs (12). During the period of two-dimensional RT and three-dimensional conformal RT (3DCRT), prediction of radiation-induced xerostomia has been frequently studied based on normal tissue complication probability (NTCP) models depending on the dose–volume relationship with the probability of side effects, using either a univariate or a multivariate logistic regression model (10, 13, 14). However, not only dose–volume parameters but also other clinical prognostic factors could affect radiation-induced xerostomia. A multivariable logistic regression model needs to be developed to take a wide variety of influencing factors into consideration. The least absolute shrinkage and selection operator (LASSO) is a relatively refined model that constructs a penalty function so that some regression coefficients are compressed. That is, the sum of absolute values of the mandatory coefficients is less than a fixed value; meanwhile, some regression coefficients are set to zero. Therefore, it retains the advantage of subset contraction and is a biased estimate for processing data with complex collinearity (15). Xu et al. (16) introduced LASSO to build NTCP models of xerostomia in patients with HNC treated using 3DCRT. Lee et al. (17) reported that using a multivariate regression model with LASSO could predict the incidence of xerostomia after IMRT in patients with HNC. However, the major weakness of these studies is the lack of assessment of radiation dose to other salivary glands, including SMG and OC.

This study aimed to develop a LASSO-based multivariable NTCP model to predict radiation-induced xerostomia in patients with NPC treated using comprehensive salivary gland–sparing HT technique and to identify clinical and dosimetric factors associated with xerostomia. This study is novel in studying the probability and severity of xerostomia in a large consecutive clinical sample of patients with NPC treated with comprehensive salivary gland–sparing HT technique.

## Methods and Materials

### Participants and Data Collection

Data from 220 consecutive patients with histologically-confirmed NPC treated with comprehensive salivary gland–sparing HT technique from February 2016 to August 2018 were collected from the Department of Radiotherapy in the First Medical Center of the General Hospital of the Chinese People’s Liberation Army (PLA). Seventeen patients died from progression of the disease or other complications within the first two years after RT. The clinical characteristics of the remaining 203 patients are shown in **Table 1**. All eligible patients participated in the saliva flow rate measurement and the xerostomia questionnaire (XQ) evaluation. Data on the risk factors of xerostomia, such as age, gender, PG Dmean (total), SMG Dmean (total), OC Dmean, treatment options, T stage and N stage, saliva flow rates, and XQ score, were collected for each patient. All patients provided written informed consent. This prospective study was registered with the number ChiCTR-ONN-17010597 in the Chinese Clinical Trial Registry and was conducted at our study center and approved by the ethics committee of the Chinese PLA General Hospital (approved no. S2016-122-01).

**Table 1:** Patients’ characteristics.

Characteristics	No. of patients	%
Age (year)		
Mean	48	
Median	51	
Range	10 - 83	
Gender		
Male	138	67.98
Female	65	32.02
Treatment		
-1 (Induction chemotherapy+ concurrent chemoradiotherapy+ molecular targeted therapy)	95	46.80
-2 (Induction chemotherapy+ concurrent chemoradiotherapy)	100	49.26
-3 (Concurrent chemoradiotherapy)	3	1.48
-4 (Radiation therapy alone)	5	2.46
PG dose (Gy)		
Mean	30.15	
Median	30.05	
Range	11.19 - 43.19	
SMG dose (Gy)		
Mean	41.74	
Median	42.13	
Range	10.29 - 66.63	
OC dose (Gy)		
Mean	32.01	
Median	31.91	
Range	13.55 – 52.48	
T-stage		
T1	19	9.36
T2	99	48.77
T3	50	24.63
T4	35	17.24
N-stage		
N0	13	6.40
N1	44	21.67
N2	101	49.75
N3	45	22.17
UICC/AJCC stage		
I	2	0.99
II	33	16.26
III	94	46.31
IVa	74	36.45

PG, parotid gland; SMG, submandibular gland; OC, accessory salivary glands in the oral cavity.

### Treatment and Xerostomia Evaluation

**Figure 1:**
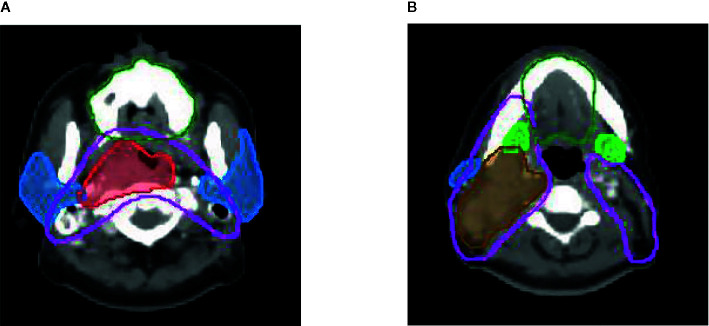
Delineation of target volume Red line: pGTVnx; brown line: pGTVnd; pink line: PTV1; dark green line: oral cavity; blue line: parotid gland; green line: submandibular gland.

All patients were treated with comprehensive salivary gland–sparing HT technique. The prescription dose to the primary tumor and metastatic lymph nodes was 67.5 Gy, accompanied with 60 Gy to high-risk areas and 54 Gy to low-risk areas, in 30 fractions. The mean doses were constrained to be as low as possible for PG, SMG, and OC, while the dose to target areas was not compromised with the relevant salivary gland protection. Target volumes were delineated, as shown in **Figure 1**. IMRT was performed using 6-MV x-ray obtained using a TomoTherapy System (Accuray, USA). The main treatment model was induction chemotherapy, followed by concurrent chemoradiotherapy. On this basis, weekly Nituzumab was added to concurrent chemoradiotherapy in some patients. Xerostomia was evaluated by a questionnaire and saliva flow rate measurement before RT and at 0, 1, 3, 6, 12, 18, and 24 months after the end of RT. The xerostomia-specific questionnaire was tested and validated (8, 18). Saliva flow rates, including unstimulated and stimulated saliva flow rates, were measured as reported in a previous study (8).

### Statistical Analyses

Descriptive statistics were presented in the baseline characteristics table. As bilateral glands were exposed to different doses in patients with different clinical stages, the average of the Dmean of both PGs and SMGs was calculated for the convenience of analysis. Potential prediction variables, including age, gender, PG Dmean, SMG Dmean, OC Dmean, treatment options, T stage, N stage, UICC/AJCC stage, saliva flow rates, and XQ score, were analyzed by multivariate linear regression. Treatment, T stage, N stage, and AJCC stage are categorical variables. In the prediction model, one of the variables is selected as the reference point to analyze its correlation with the other variables. The Mann−Kendall trend test was used to verify the consistency of the XQ score and saliva flow rates. Statistical comparisons of continuous variables were performed using the independent-samples *t* test or Mann–Whitney *U* test for the two groups. Categorical variables were expressed as percentages, and statistical comparisons were performed using the *X* ^2^ test or Fisher’s exact test. All statistical tests were performed using R (version 4.0.2) statistical software, and a two-sided *P <*0.05 was deemed to be statistically significant.

### Prediction Model

As the dependent variable was one (change) or zero (unchange) for the predictive factors affecting xerostomia at 1-year or 2-year postradiotherapy, logistic regression with an extended bootstrapping technique was used, which was defined as follows:

$$P=\frac{1}{1+e^{-A}}$$

Here, *P* represents the alleviation probability of the radiation-induced xerostomia. *A* = *β*_0_ + *β*_1_ *X*_11_ + *β*_2_ *X*_22_ +···+*β_p_X_pp_*, where *β*_0_ is the intercept term, *p* is the number of variables, *X*_11_,*X*_22_,···,*X_pp_* represent different variables, and *β*_1_,*β*_2_,···,*β_p_* represent the corresponding regression coefficient. Maximum likelihood estimation was adopted in the parameter estimation process. Two models were constructed according to the patient-reported XQ score, which were relieved by 50% and 80% compared with the level at the end of RT within 1 and 2 years, respectively. In this study, R50 and R80 were used to represent patient-reported XQ scores relieved by 50% and 80%, respectively, compared with the level at the end of RT. The dependent variables were R50 or R80 within 1 and 2 years, and independent variables were gender, age, PG Dmean, SMG Dmean, OC Dmean, T stage, N stage, UICC/AJCC stage, and treatment options. For each NPC patient, nine candidate prognostic factors were initially evaluated in the variable selection procedure. The LASSO-based multivariable NTCP model was used to predict radiation-induced xerostomia in patients with NPC treated with comprehensive salivary gland–sparing HT technique. First, the LASSO was used to rank the correlations of different potential prognostic factors, and a bootstrapping method was used to reduce the number of factors. After selecting the prognostic factors, odds ratios and 95% confidence intervals (95% CIs) were calculated for these factors (16).

Double cross-validation was carried out using training data and validation data to develop the NTCP model and test its prediction power. A model could be developed and optimized by a training set and a validation set, while the prediction power of this model was tested by a test set (17, 19). In practice, a 10-fold approach is used more often, and the prediction likelihood of 10-fold cross-validation is relatively stable, as reported by Xu et al. (17). Therefore, in this study, 10-fold cross-validation was used to obtain the best predictive factor subsets. The area under the receiver operating characteristic curve (AUC) was also used as another criterion to check the performance of the model (20).

## Results

### Patients

A total of 203 patients were enrolled in this study (**Table 1**). Patients were predominantly male (67.98%), with a median age of 51 years (10–83 years). Patients, with stage II (16.26%), III (46.31%), and IVa (36.45%), received induction chemotherapy combined with concurrent chemoradiotherapy (49.26%), and induction chemotherapy combined with concurrent chemoradiotherapy and Nituzumab (46.80%). The doses were constrained to be as low as possible following IMRT by helical tomotherapy technique for bilateral PG (PG-T, with the average doses of both glands), contralateral SMG (cSMG), and OC, with an average of the mean dose of these glands of 30.15Gy (range from 11.19 to 43.19Gy), 41.74Gy (range from 10.29 to 66.63Gy), and 32.01Gy (range from 13.55 to 52.88Gy), respectively. The median time from therapy to the last follow-up was 44 months (25–54 months).

### Consistency Between XQ Evaluation and Saliva Flow Rate Measurement

A strong consistency between the XQ score and saliva flow rates was detected by the Mann−Kendall trend with *P <*0.05 in 159 cases, accounting for 78.33% of the cases with unstimulated saliva flow rate measurement, while with *P <*0.05 in 161 cases, accounting for 79.31% of the cases with stimulated saliva flow rate measurement (**Supplementary Table 1**). Therefore, the XQ score was used to evaluate xerostomia in the subsequent analyses.

### Correlation of Different Predictive Factors

**Figure 2:**
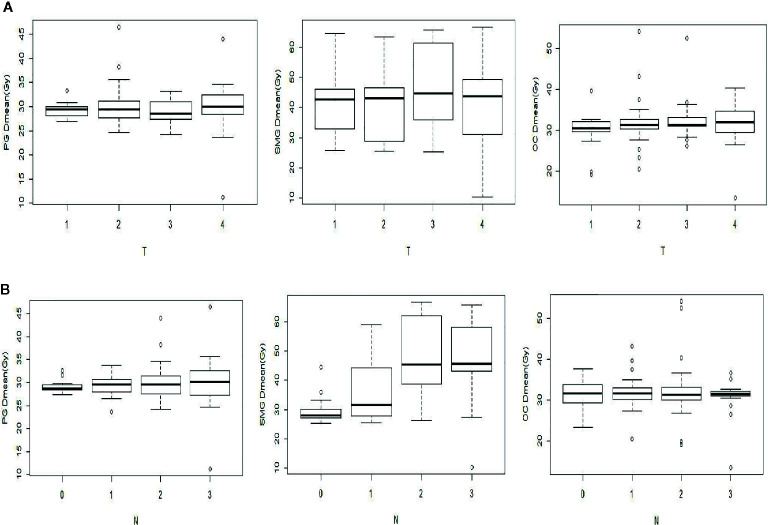
Comparison of the mean dose (Dmean) of PG, SMG, and OC with different T **(A)** and N **(B)** stages.

As shown in **Figure 2A**, SMG Dmean changed significantly with different T stages, which had no significant effect on the PG Dmean or OC Dmean. However, different N stages had a significant effect on the SMG Dmean, which was about 1.5 times higher in patients with N2–3 stages than in those with N0–1 stage (**Figure 2B**).

### Predictors of R50 or R80 at 1-Year and 2-Year Postradiotherapy

The factors that correlated with the patient-reported XQ score, at 12- and 24-month postradiotherapy, detected by univariate and multivariate analyses are summarized in **Table 2**. At 1-year postradiotherapy, age, gender, and SMG Dmean each significantly correlated with R50 in the multivariate model, while no factors correlated with R50 at 2-year postradiotherapy. Furthermore, at 1-year postradiotherapy, just age and OC Dmean correlated with R80, whereas age, gender, SMG Dmean, and OC Dmean correlated with R80 at 2-year postradiotherapy. **Supplementary Figure 1** shows that the recovery probability of xerostomia represented by R50 and R80 increased with a prolonged follow-up. The R50/R80 returned to 69.95%/6.40% at 12 months and to 95.57%/66.01% at 24 months, respectively. That is to say, at 1-year postradiotherapy, very few patients reached the R80 level. However, almost all the patients reached the R50 level at 2-year postradiotherapy. Therefore, the probability of R50 at 1-year postradiotherapy (R50-1year) and the probability of R80 at 2-year postradiotherapy (R80-2years) were finally chosen to establish the NTCP model for radiation-induced xerostomia.

**Table 2:** Predictors of R50/R80 at 1 year and 2 years of post-radiotherapy.

Variable	1-year Multivariate model		2-years Multivariate model	
	R50	R80	R50	R80
**Age**	-2.52* (1.03)	-6.93** (2.20)	-3.23 (3.72)	-7.06***(1.51)
**Gender**	1.34***(0.40)	0.86 (2.20)	3.27 (1.53)	2.96***(0.61)
**OC Dmean**	-0.06 (0.06)	0.17* (0.12)	-0.14 (0.15)	-0.18*(0.09)
**PG Dmean**	0.30 (2.52)	2.61 (5.30)	-5.72 (9.63)	0.60 (3.17)
**SMG Dmean**	-0.11***(0.02)	-0.09 (0.04)	-0.43 (0.26)	-0.22***(0.04)
**Treatment**				
** Treatment-1**				
** Treatment-2**	0.20 (0.42)	1.43 (0.87)	-0.39 (1.46)	-0.30 (0.56)
** Treatment-3**	14.78 (1172.40)	-16.94 (11500)	13.78 (22160)	14.15 (3355.10)
** Treatment-4**	-0.70 (1.40)	-10.16 (3920)	-6.37 (4.44)	2.20 (1.88)
**T stage**				
** T1**				
** T2**	-0.51 (0.72)	16.70 (2090)	-13.66 (8462)	1.03 (0.87)
** T3**	-0.41 (0.83)	16.22 (2090)	-12.41 (8462)	1.48 (1.05)
** T4**	-0.88 (1.40)	13.49 (2090)	-30.89 (9647)	0.53 (1.40)
**N stage**				
** N0**				
** N1**	0.19 (1.06)	17.30 (2930)	-11.67 (11357)	-15.43 (1576.11)
** N2**	1.17 (1.15)	15.04 (2930)	-4.68 (11357)	-16.64 (1576.11)
** N3**	2.54 (1.66)	15.30 (2930)	7.64 (12218)	-16.82 (1576.11)
**UICC/AJCC stage**				
** I**				
** II**	-14.73 (1392.27)	-15.49 (13800)	23.99 (33030)	0.78 (4244.68)
** III**	-15.20 (1392.27)	-13.63 (13800)	4.74 (32452)	2.76 (4244.68)
** IV**	-16.62 (1392.27)	-12.08 (13800)	26.02 (32781)	2.97 (4244.68)
**(Constant)**	22.53 (1392.27)	-23.25 (137000)	50.52 (29198)	30.70 (3941.22)

R50/R80, patient-reported xerostomia scores relieved by 50%/80% compared to the level at the end of radiation therapy. *p = 0.05; **p = 0.01; ***p = 0.001.

### Prediction Model With R50-1year and R80-2years

**Figure 3:**
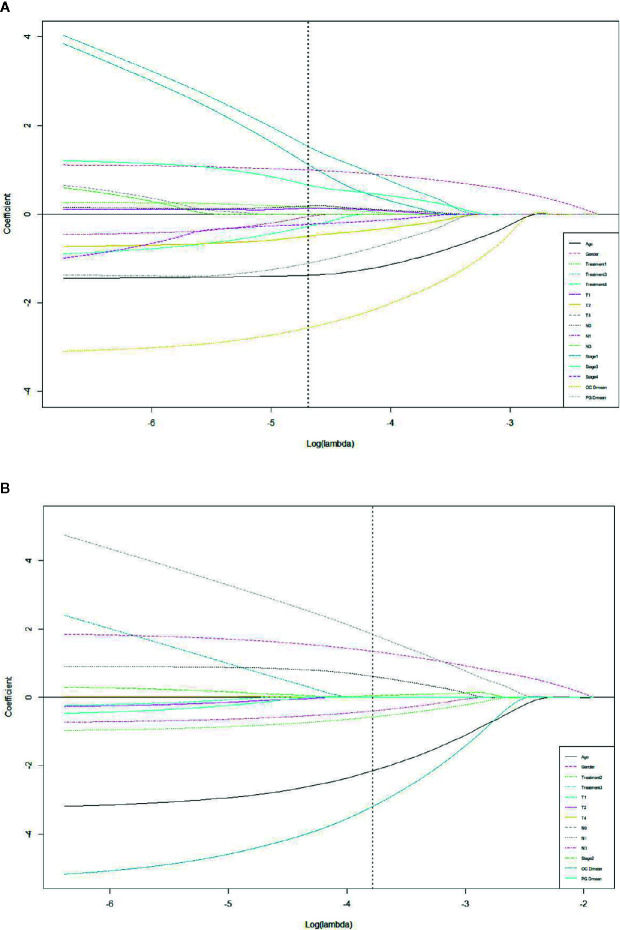
LASSO coefficient profiles of the eleven R50-1year **(A)** and sixteen R80-2years **(B)** related events with non-zero coefficients determined by the optimal lambda.

LASSO with bootstrap technique ranked the predictive factors of R50-1year and R80-2years in descending order, as shown in **Supplementary Table 2**. The 10-fold cross-validation was used to test the prediction performance of NTCP models. The LASSO coefficient profiles of the R50-1year and R80-2years with nonzero coefficients determined by the optimal lambda (λ) are shown in **Figures 3A, B**. λ is the regularization parameter in LASSO, and the optimal value could be obtained from the 10-fold cross-validation. When log (λ) = −4.7, seven predictive factors of R50-1year were selected: age, gender, T stage, UICC/AJCC stage, PG Dmean, OC Dmean, and treatment options. When log(λ) = −3.8, six prognostic factors of R80-2years were selected: age, gender, T stage, UICC/AJCC stage, OC Dmean, and N stage. All corresponding coefficients of the multivariate logistic regression models are shown in **Tables 3** and **4**. The probability of xerostomia recovery in each patient could be calculated using the following formula:

$$P=\frac{1}{1+e^{-A}}$$

**Table 3:** Multivariate logistic regression in the R50-1year model for optimal prediction factors selection.

Prognostic factor (p= 7)	*β*	p	Odds ratio	95% CI
**Age**	-1.45	0.111	0.23	0.04 - 1.35
**Gender**	1.13	0.001	3.10	1.57 - 6.23
**Treatment**				
** Treatment-1**	0	<0.001		
** Treatment-2**	-0.27	0.431	0.76	0.38 - 1.50
** Treatment-3**	15.36	0.989	4665963	
** Treatment-4**	-1.28	0.321	0.28	0.02 - 3.64
**T stage**				
** T1**	0	<0.001		
** T2**	-0.86	0.183	0.42	0.10 - 1.40
** T3**	-0.10	0.889	0.90	0.20-3.71
** T4**	-0.88	0.499	2.42	0.23 - 59.06
**OC Dmean**	-0.08	0.081	0.92	0.84 - 1.01
**PG Dmean**	-0.04	0.487	0.96	0.85 - 1.08
**UICC/AJCC stage**				
** I**	0	<0.001		
** II**	-14.35	0.991	0.00	
** III**	-15.62	0.991	0.00	
** IV**	-16.98	0.990	0.00	
**(Constant)**	21.28	0.987		

**Table 4:** Multivariate logistic regression in the R80-2years model for optimal prediction factors selection.

Prognostic factor (p= 6)	*β*	p	Odds ratio	95% CI
**Age**	-3.45	0.001	0.03	0 - 0.21
**Gender**	1.92	0.000	6.79	3.20 - 15.27
**Treatment**				
** Treatment-1**	0	<0.001		
** Treatment-2**	-1.00	0.007	0.37	0.17 - 0.75
** Treatment-3**	15.23	0.994	4123962	
** Treatment-4**	0.19	0.900	1.22	0.04-37.83
**OC Dmean**	-0.14	0.006	0.87	0.78 - 0.95
**UICC/AJCC stage**				
** I**	0	<0.001		
** II**	0.95	1.000	2.58	
** III**	0.84	1.000	2.32	0.33 - 14.43
** IV**	0.98	1.000	2.67	0.22 - 76.48
**N stage**				
** N0**	0	<0.001		
** N1**	-16.20	0.988	0	
** N2**	-17.27	0.987	0	
** N3**	-18.17	0.987	0	
**(Constant)**	22.51	0.992	5975743863	

In the R50-1year model, *A* = 3.52 − (age × 1.45) + (gender × 1.13) + (treatment × corresponding coefficient) + (T stage × corresponding coefficient) − (PG Dmean × 1.36) − (OC Dmean × 3.14) + (UICC/AJCC stage × corresponding coefficient). In the R80-2years model, *A* = 2.87 − (age × 2.25) + (gender × 1.38) + (treatment × corresponding coefficient) − (OC Dmean × 3.36) + (UICC/AJCC stage × corresponding coefficient) + (N stage × corresponding coefficient).

The AUC of the forward selection model was achieved through 200 randomized LASSO tests; the average was 0.72 (95% CI = 0.56–0.87) for the R50-1year model, while 0.82 (95% CI = 0.70–0.95) for the R80-2years model.

## Discussion

Xerostomia is one of the most common RT-induced toxicities in patients with NPC (10, 19). Identifying the relevant factors and establishing a prediction model is crucial to alleviate this side effect. At present, a LASSO-based multivariable NTCP model has been used to develop the prediction model for xerostomia (16, 21). Compared with other NTCP prediction models, this model is more suitable for multiple complex variable factors using the regularization method. A bias term was added to the regression optimization function to reduce the collinearity effect, thus reducing the model variance. Radiation-induced xerostomia usually takes a longer time to recover. However, most current models set the end point at the 12th month after RT. In addition, most of the xerostomia risk prediction models are based on the dose–volume threshold of the PG (11, 22). Although the dose and volume of the PG could be effectively reduced by IMRT technique (4, 23, 24), other salivary glands were also involved in saliva production. At present, comprehensive protection of salivary glands, including PG, SMG, and OC, has been demonstrated to significantly alleviate xerostomia in patients with HNC treated with HT, without increasing the locoregional recurrence risk (8). Other clinical prognostic factors could affect radiation-induced xerostomia. Therefore, LASSO-based multivariable NTCP models were developed to predict radiation-induced xerostomia among patients with NPC treated with comprehensive salivary gland–sparing HT technique at 1-year and 2-year postradiotherapy.

In this study, multivariate analysis showed that age, gender, and SMG Dmean were predictors of R50-1year, while age, gender, SMG Dmean, and OC Dmean were predictors of R80-2years. Therefore, not only SMG Dmean, but also age, gender, and OC Dmean were the principal predictive factors of xerostomia. This result was consistent with clinical observations and was similar to a previous study (16). The female patients had a higher probability of xerostomia than male patients, along with older patients who had a higher probability of xerostomia than younger patients. Onjukka et al. (25) recently reported that age was one of the significant variables for severe xerostomia in patients with HNC after RT. The reason might be that younger patients recover more quickly from radiation-induced gland damage. However, why women are more prone to radiation-induced xerostomia is not clear. Jellema et al. (26) reported that two-dimensional radiation-induced xerostomia had a larger impact on the overall quality of life in women than in men, and this may be because women experienced more insomnia than men (27). Further research is needed to clarify if the endocrine system and psychological factors are also involved. Saarilahti et al. (28) demonstrated that sparing of contralateral SMG resulted in a reduction of xerostomia compared with patients with only PG spared. SMG-sparing IMRT realized with HT technique had been an effective method to reduce the risk of xerostomia in patients with NPC. Although OC Dmean is a non-negligible variable, the amount of saliva secreted by the OC is relatively small, and oral discomfort is mainly caused by the mucosal injury. Eisbruch et al. (29) found that restricting the threshold of OC Dmean to 41.6 Gy in 84 patients with HNC could protect OC and reduce xerostomia symptoms. However, large sample studies are still needed to determine the relationship between oral dosimetry and xerostomia. From the multivariate analysis, not only dose–volume parameters, such as SMG Dmean, but also varieties of clinical factors were detected as risk factors for xerostomia. A LASSO-based multivariable NTCP model was built so as to take a wide variety of influencing factors into consideration. The aim of this study was to investigate the probability and severity of radiation-induced xerostomia in a large consecutive clinical sample of patients with NPC treated with comprehensive salivary gland–sparing HT technique first. Furthermore, a LASSO-based multivariable NTCP model showed superior prediction performance (improving efficiency and fitness) under the conditions of variables in the data set with high dimensions and multicollinearity. Finally, the end point of follow-up in this study was extended to 24 months.

The prediction model of R50-1year and R80-2years was achieved by LASSO using the bootstrapping method. The difference between the two models was detected because in addition to the five common predictive factors, the T stage and PG Dmean were prediction variables of R50-1year, while the N stage was the prediction variable of R80-2years. This suggested that the N stage was one of the predictive factors of xerostomia with a long follow-up. One possible explanation was that dose distribution in the neck varied with different N stages, affecting PGs and SMGs, leading to their injury in patients with advanced N stage. However, SMG Dmean was not detected as a predictive factor in the two models, probably because SMG Dmean was closely related to the N stage, and both of them might be multicollinear. The explanatory variables, such as SMG Dmean and N stage, in the regression model were distorted or difficult to estimate due to the precise correlation or high correlation. As a result, the N stage was a highly significant variable, causing SMG Dmean to change from significant to insignificant in the outcome variable in the prediction model, in which the primary goal was to improve the prediction accuracy, and multicollinearity was allowed.

In this study, 10-fold cross-validation was used to test the prediction performance of the NTCP models. After validation, the AUC index for the prediction model of R50-1year and R80-2years was 0.72 and 0.82, respectively, demonstrating a good performance of the models. The 10-fold cross-validation, more stable than 2-fold or 5-fold cross-validation, divided the data set into 10 parts and took 9 parts as the training data and 1 part as the test data, in turn, to conduct the test. The average value of the correct rate (or error rate) of the results of 10 times was used as the estimation of the accuracy of the algorithm. This study showed that 10-fold cross-validation was an appropriate choice for obtaining the best error estimate and was used as an optimization model.

This study constructed R50-1year and R80-2years by LASSO using the bootstrapping method as prediction models of radiation-induced xerostomia in patients with NPC treated with comprehensive salivary gland–sparing HT technique. However, this study was a single-institution study. As only two patients had UJCC stage 1, the sample size should be further expanded in future studies. Therefore, the prediction models might not be suitable for other centers. Furthermore, the clinical correlation variables might be insufficient, and more characteristics of patients, such as eating habits, smoking and drinking habits, place of origin, and degree of education, might be necessary to be incorporated into the construction of the prediction model.

## Conclusions

The prediction model by LASSO with 10-fold cross-validation showed that radiation-induced xerostomia could be predicted by prognostic factors of R50-1year (age, gender, T stage, UICC/AJCC stage, PG Dmean, OC Dmean, and treatment options) and R80-2year (age, gender, UICC/AJCC stage, OC Dmean, N stage, and treatment options) with a good performance by the AUC. Therefore, these two models are recommended to validate the NTCP models before comprehensive salivary gland–sparing RT in patients with NPC.

## Data Availability Statement

The original contributions presented in the study are included in the article/**Supplementary Material**. Further inquiries can be directed to the corresponding authors.

## Ethics Statement

This prospective study registered with number ChiCTR-ONN-17010597 in Chinese Clinical Trial Registry was conducted in our center and approved by the Ethics Committee of the Chinese PLA General Hospital (approved no. S2016-122-01). Written informed consent to participate in this study was provided by the participants’ legal guardian/next of kin.

## Author Contributions

FT and WF contributed equally to this work, participated in the design of the study, carried out the study, and drafted the manuscript. YL, SX, HG, RG, and XZ helped to carry out the study. XW reviewed the manuscript and performed the statistical analysis. LM conceived and designed the study and edited and reviewed the manuscript. All authors read and approved the final manuscript. All authors contributed to the article and approved the submitted version.

## Conflict of Interest

The authors declare that the research was conducted in the absence of any commercial or financial relationships that could be construed as a potential conflict of interest.
