# Supplementary material for: A Risk Prediction Model by LASSO for Radiation-Induced Xerostomia in Patients With Nasopharyngeal Carcinoma Treated With Comprehensive Salivary Gland–Sparing Helical Tomotherapy Technique
Source: Front Oncol. 2021 Feb 26;11:633556. doi: 10.3389/fonc.2021.633556 (PMC7953987; doi:10.3389/fonc.2021.633556)
Supplement: Supplementary file 1 [file DataSheet_1.docx]

**Table 1. Consistency of the XQ score with saliva flow rates measurement**

| **Mann-Kendall trend test (*p* value)** | **USFR No. (%)** | **SFR No. (%)** |
| --- | --- | --- |
| ＜0.05 | 159（78.33%） | 161（79.31%） |
| ≥0.05 | 44（21.67%） | 42（20.69%） |

XQ: xerostomia questionnaire; USFR: Unstimulated saliva flow rates; SFR: Stimulated saliva flow rates

**Table 2.** **Prognostic factors correlation ranking for R50-1year and R80-2years by LASSO**

| **R50-1year** |  |  |  |  |
| --- | --- | --- | --- | --- |
| 1. SMG Dmean | 2. Gender | 3. Age | 4. OC Dmean | 5. AJCC stage |
| 6. PG Dmean | 7. N stage | 8. Treatment | 9. T stage |  |
| **R80-2years** |  |  |  |  |
| 1. SMG Dmean | 2. Gender | 3. N stage | 4. Age | 5. OC Dmean |
| 6. AJCC stage | 7. T stage | 8. PG Dmean | 9. Treatment |  |

R50-1year: patient-reported xerostomia scores relieved by 50% compared to the level at the end of radiotherapy at 1 year; R80-2years: patient-reported xerostomia scores relieved by 80% compared to the level at the end of radiotherapy at 2 years.


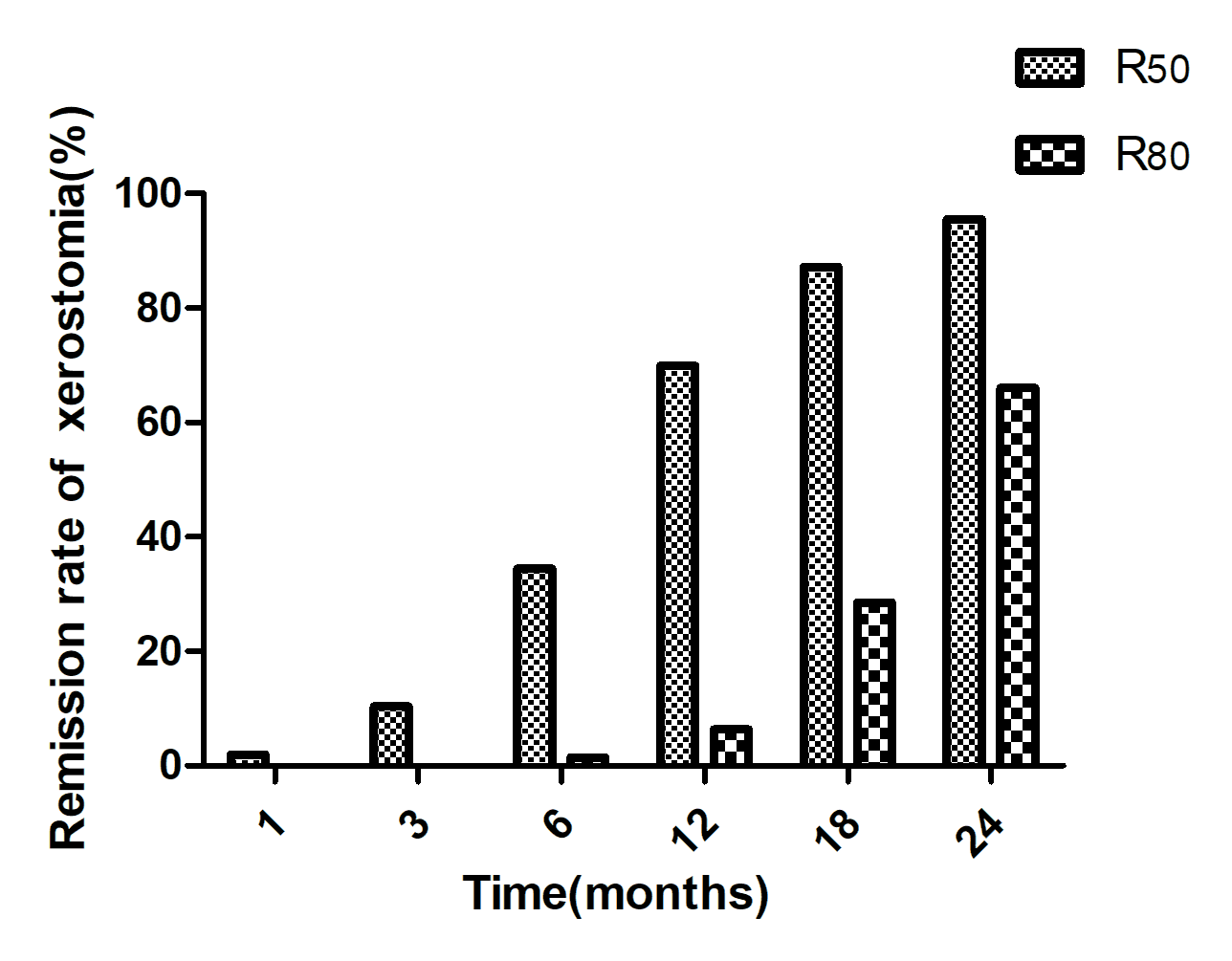


**Figure 1. R50 versus R80 at different post-radiotherapy time points (1, 3, 6, 12, 18 and 24 months)**

R50/R80: patient-reported xerostomia scores relieved by 50%/80% compared to the level at the end of radiation therapy.
